# Aggregation behaviour of a single-chain, phenylene-modified bolalipid and its miscibility with classical phospholipids

**DOI:** 10.3762/bjoc.13.99

**Published:** 2017-05-23

**Authors:** Simon Drescher, Vasil M Garamus, Christopher J Garvey, Annette Meister, Alfred Blume

**Affiliations:** 1 Institute of Pharmacy, Martin Luther University (MLU) Halle-Wittenberg, Wolfgang-Langenbeck-Str. 4, 06120 Halle (Saale), Germany; 2 Helmholtz-Zentrum Geesthacht (HZG), Centre for Materials and Costal Research, Max-Planck-Str. 1, 21502 Geesthacht, Germany; 3 Australian Nuclear Science and Technology Organisation (ANSTO), Kirrawee DC, NSW, Australia; 4 Institute of Chemistry, MLU Halle-Wittenberg, von-Danckelmann-Platz 4, 06120 Halle (Saale), Germany; 5 Institute of Biochemistry and Biotechnology, MLU Halle-Wittenberg, Kurt-Mothes-Str. 3, 06120 Halle (Saale), Germany

**Keywords:** aggregation behaviour, bolaamphiphiles, bolalipids, membrane lipids, mixing behaviour, nanofibres, self-assembly

## Abstract

In the present work, we describe the synthesis of a single-chain, phenylene-modified bolalipid with two phosphocholine headgroups, PC-C18pPhC18-PC, using a Sonogashira cross-coupling reaction as a key step. The aggregation behaviour was studied as a function of temperature using transmission electron microscopy (TEM), differential scanning calorimetry (DSC), Fourier-transform infrared (FTIR) spectroscopy, and small angle neutron scattering (SANS). We show that our new bolalipid self-assembles into nanofibres, which transform into flexible nanofibres at 27 °C and further to small elongated micelles at 45 °C. Furthermore, the miscibility of the bolalipid with bilayer-forming phosphatidylcholines (DMPC, DPPC, and DSPC) was investigated by means of DSC, TEM, FTIR, and small angle X-ray scattering (SAXS). We could show that the PC-C18pPhC18-PC is partially miscible with saturated phosphatidylcholines; however, closed lipid vesicles with an increased thermal stability were not found. Instead, bilayer fragments and disk-like aggregates are formed.

## Introduction

Bolalipids are amphiphilic molecules consisting of two hydrophilic headgroups attached to both ends of a long hydrocarbon spacer [1]. The hydrophobic spacer is composed of either a single alkyl chain or two chains connected via a glycerol moiety. These bolalipids originate in membranes of some species of archaea, e.g., thermoacidophiles and these archaeal membranes can withstand extreme living conditions, such as high temperatures or low pH values [2–4]. Archaea are quite different from bacteria and eukaryotes [5–9], which is also reflected in the chemical structure of those archaeal membrane lipids: the alkyl chains are connected via ether linkages in the inverse *sn*-2,3 configuration to the glycerol, the alkyl chains sometimes contain a varying number of cyclopentane rings or several methyl branches [2–3], and some of the archaeal lipids consist of two transmembrane alkyl chains (caldarchaeol-type). Especially this type of bolalipids is of great interest for applications in material sciences, biotechnology, and pharmaceuticals [10–15]. Since these bolalipids are able to span the membrane of classical phospholipid bilayers, they can be used to stabilize liposomes for drug delivery purposes. The applicability of this approach was already tested for a large variety of natural and artificial bolalipids [12,16–22].

The isolation of archaeal bolalipids from natural sources is expensive and often leads to mixtures of bolalipids with different alkyl chain pattern. But also the synthesis of natural as well as artificial bolalipids is elaborate and time-consuming and, hence, present research tries to simplify the chemical structure of bolalipids by keeping up their membrane-stabilizing properties [23]. This simplification strategy led in our group to the synthesis of dotriacontane-1,32-diylbis[2-(trimethylammonio)ethylphosphate] (PC-C32-PC) [24–25], the simplest bola model lipid consisting of two phosphocholine (PC) headgroups connected by a long, unmodified C32 alkyl chain. If PC-C32-PC is suspended in water, the formation of a dense network of nanofibres and, as a consequence, a clear and transparent hydrogel is observed [25]. The nanofibres have a thickness of about 6 nm, corresponding to the length of a PC-C32-PC molecule. Due to the bulky PC headgroup, the PC-C32-PC molecules are arranged side by side within the fibrous aggregate but slightly twisted relative to each other leading to a helical super structure of the fibres. This helicity was previously confirmed by cryo-transmission electron microscopy (cryo-TEM), high resolution atomic force microscopy (AFM) [26], and Monte Carlo simulations [27]. A temperature increase leads to a transformation of the nanofibres into small micelles and the gel character is lost. This reversible gel/sol transformation is accompanied by a cooperative endothermic transition at *T*_m_ = 48 °C, which can be followed by differential scanning calorimetry (DSC) [24].

However, the use of PC-C32-PC as “stabilizer” of phospholipid bilayers failed. In mixtures of PC-C32-PC with classical phospholipids, such as 1,2-dipalmitoyl-*sn*-glycero-3-phosphocholine (DPPC) or 1-palmitoyl-2-oleoyl-*sn*-glycero-3-phosphocholine (POPC), no significant insertion of the bolalipid into the bilayer was observed [28]. The reason for this behaviour is that packing problems due to the mismatch between the large space requirement of the PC headgroup of PC-C32-PC and the small cross-sectional area of its single alkyl chain arise. The insertion of a PC-C32-PC molecule in a stretched conformation into phospholipid bilayers is energetically unfavourable as it produces void volume, which can be filled by neither bolalipid nor phospholipid. Consequently, the self-assembly of PC-C32-PC into nanofibres is preferred.

**Figure 1:**
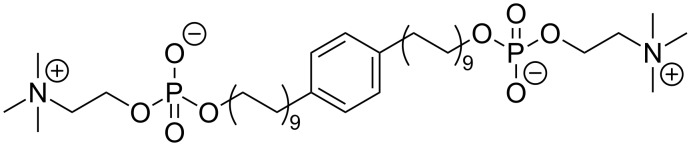
Chemical structure of PC-C18pPhC18-PC.

To evade these packing problems, we expanded the cross-sectional area of the alkyl chain of the bolalipid in order to fill the void volume. Besides heteroatoms [29–30], acetylene [31] or diacetylene groups [32], or methyl branches [31,33], also phenyl- or biphenyl rings were inserted into the long alkyl chain [34–37]. The insertion of a phenylene group led to PC-C16pPhC16-PC [36], which self-assembles at room temperature into small ellipsoidal micelles, and to PC-C17pPhC17-PC [35] that forms nanofibres with a significantly reduced thermal stability compared to PC-C32-PC. Unfortunately, the insertion of PC-C17pPhC17-PC into phospholipid bilayers composed of, e.g*.*, DPPC did not result in the formation of stabilized lipid vesicles and elongated micelles as well as bilayer fragments were found instead. We have now synthesized a bolalipid with phenyl modification and a slightly longer alkyl chain, PC-C18pPhC18-PC (Figure 1), to investigate a possible chain length dependency on both the aggregation behaviour of the pure bolalipid and the miscibility with bilayer-forming phospholipids.

In this study, we investigated the aggregation behaviour of PC-C18pPhC18-PC in aqueous suspension by means of DSC, TEM, Fourier-transform infrared (FTIR) spectroscopy, and small angle neutron scattering (SANS). Moreover, the mixing behaviour of PC-C18pPhC18-PC with saturated phosphatidylcholines 1,2-dimyristoyl-*sn*-glycero-3-phosphocholine (DMPC), DPPC, and 1,2-distearoyl-*sn*-glycero-3-phosphocholine (DSPC) was studied by means of DSC and TEM. Additionally, one mixture was exemplarily investigated by FTIR and small angle X-ray scattering (SAXS).

## Results and Discussion

### Synthesis of PC-C18pPhC18-PC and its temperature-dependent aggregation behaviour

#### Synthesis

**Scheme 1:**
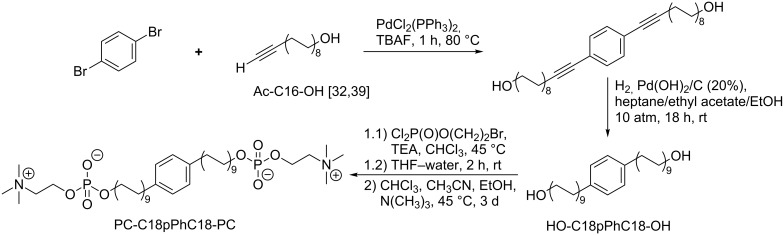
Synthesis of PC-C18pPhC18-PC; TBAF: tetra-*n*-butylammonium fluoride, TEA: triethylamine, rt: room temperature.

The phenylene-modified bolalipid 18,18’-(1,4-phenylene)bis{octadec-1-yl[2-(trimethylammonio)ethylphosphate]} (PC-C18pPhC18-PC) was synthesised from the corresponding diol (HO-C18pPhC18-OH) by established phosphorylation and quarternisation reactions described previously [38]. The long-chain, phenylene-modified 1,ω-diol in turn was prepared using a bis-Sonogashira cross-coupling reaction [37] with PdCl_2_(PPh_3_)_2_ as catalyst and tetra-*n*-butylammonium fluoride (TBAF) as solvent as well as 1,4-dibromobenzene and octadec-17-yn-1-ol (Ac-C16-OH) [32,39] as starting material. Following, both triple bonds were converted into single bonds by hydrogenation. The bolalipid was finally purified by middle pressure liquid chromatography (MPLC) using CHCl_3_/MeOH/H_2_O as eluent and the gradient technique. The synthetic pathway is summarised in Scheme 1. Details of synthetic procedures and analytical data can be found in Supporting Information File 1.

#### Aggregation behaviour

The aggregation behaviour of this novel bolalipid was investigated as a function of temperature by means of TEM, DSC, FTIR, and SANS. The results obtained were compared to PC-C17pPhC17-PC [35] and PC-C16pPhC16-PC [36], structural analogues with slightly shorter alkyl chains, and the homologous series of phenylene-free PC-Cn-PC, with *n* = 22–36 [24–26,38,40].

The first observation is a gelation of the suspension when the bolalipid PC-C18pPhC18-PC is dissolved in water at a concentration of *c* = 1 mg mL^–1^. This behaviour indicates the formation of nanofibres, which in turn immobilize the solvent molecules and allow the formation of a transparent hydrogel. A similar observation was found for PC-C17pPhC17-PC [35] and also for phenylene-free analogues, such as PC-Cn-PC with alkyl chain lengths ranging from *n* = 22–36 [24–26,38,40].

#### DSC and FTIR

**Figure 2:**
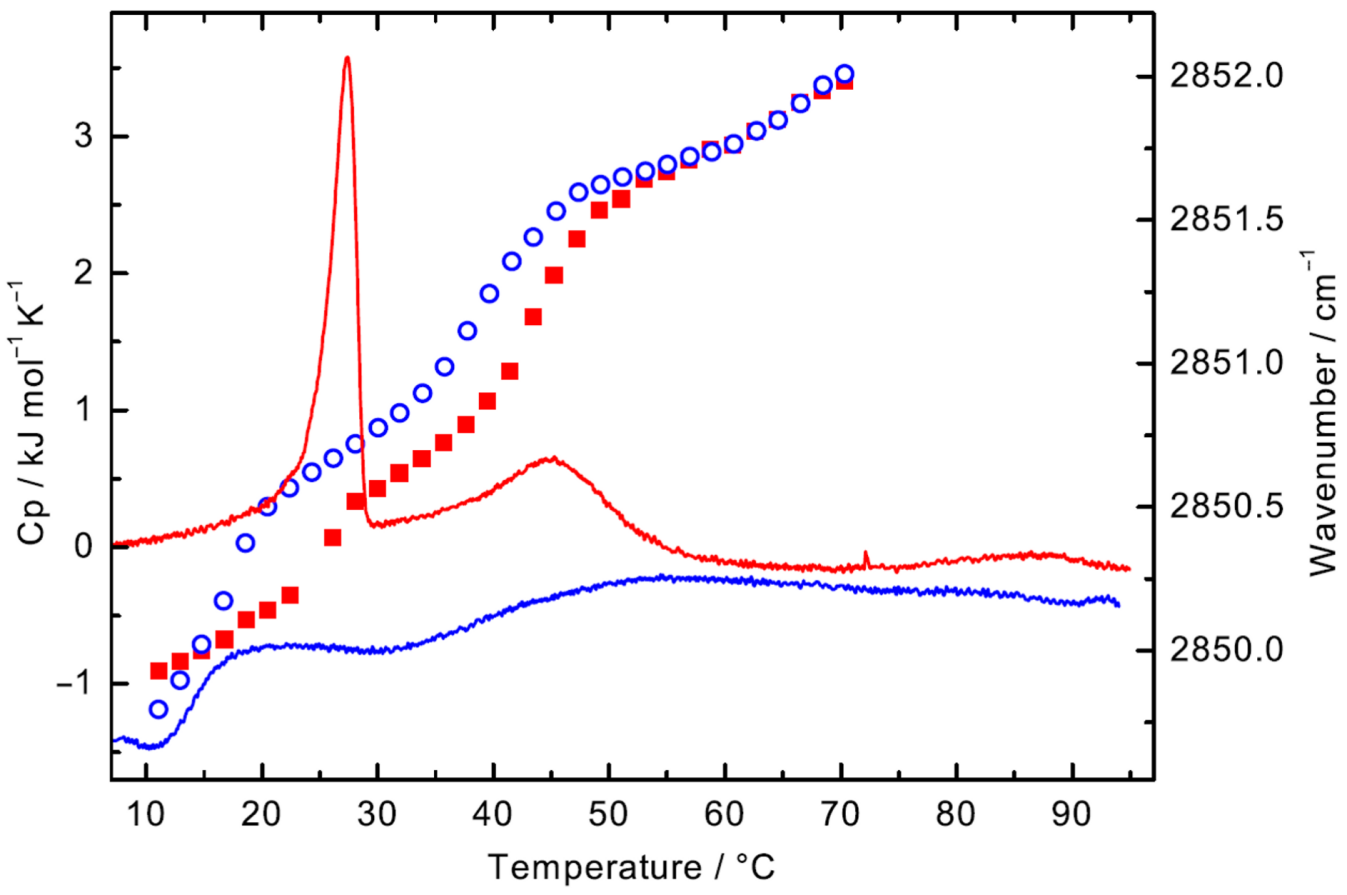
DSC curves for PC-C18pPhC18-PC (*c* = 1 mg mL^−1^ in H_2_O, solid lines, heating: red, cooling: blue). FTIR: wavenumber of the symmetric methylene stretching vibration (symbols, *c* = 50 mg mL^−1^ in H_2_O, heating: filled red squares, cooling: open blue circles).

The DSC heating curve of PC-C18pPhC18-PC (*c* = 1 mg mL^−1^ in H_2_O) shows two endothermic transitions (Figure 2): the first one at *T*_m_ = 27.4 °C (∆*H* ≈ 12 kJ mol^−1^) and a second broad peak at about 45 °C (∆*H* ≈ 6 kJ mol^–1^). The corresponding cooling curve depicts also two but very broad peaks with a hysteresis, which is an indication for a hindered reorganisation of bola molecules within the aggregates.

Both transition temperatures in the DSC heating scan are about 5 K higher compared to *T*_m_-values of the lipid analogue PC-C17pPhC17-PC with a slightly shorter alkyl chain (two methylene units) [35]. The increase in *T*_m_ is caused by the elongation of the alkyl chain, which leads to an increase in van-der-Waals contacts of neighbouring bola molecules. This effect has been observed before with other bolalipids [38]. When the alkyl chain becomes too short, as in PC-C16pPhC16-PC whose alkyl chain is again two methylene units shorter, no fibre formation is observed but the bolalipid molecules self-assemble at room temperature into small micellar aggregates. Consequently, no endothermic transition was observed between 2–95 °C for this bolalipid [31,35–36].

To obtain information on the conformation and the mobility of the alkyl chain of PC-C18pPhC18-PC molecules within the aggregates, IR measurements of a bolalipid suspension (*c* = 50 mg mL^–1^ in H_2_O) were conducted. The position of the symmetric methylene stretching vibrational band (*ν*_s_CH_2_) gives information about the order of the alkyl chain, whether *gauche* conformers are present or not [41–42]. The wavenumber of the *ν*_s_(CH_2_) vibrational band as a function of temperature is shown in Figure 2.

At *T* = 11.1 °C, the frequency of *ν*_s_(CH_2_) is at 2849.9 cm^–1^ indicating ordered alkyl chains in all-*trans* conformation. Within the temperature range of the first transition observed in DSC, the wavenumber of this band increases to 2850.6 cm^–1^ at *T* = 30.0 °C, which is attributed to a slightly increased amount of *gauche* conformers and a more flexible alkyl chain. However, this increase in wavenumber is not as pronounced as for PC-C17pPhC17-PC within its main DSC transition, where the wavenumber of *ν*_s_(CH_2_) jumps to 2851.5 cm^–1^ [35]. If we compare the increase in frequency of *ν*_s_(CH_2_) of phenylene-free bolalipids at their first DSC transition, e.g*.,* PC-C32-PC [38] (2849.5 cm^−1^ → 2851.3 cm^−1^; fibres → micelles) and PC-C34-PC [40] (2849.6 cm^−1^ → 2850.8 cm^−1^; fibres → fibres), the increase is in our case relatively small and comparable to PC-C34-PC. This shows that the chain order in the intermediate structures of PC-C18pPhC18-PC present at 30 °C, between both DSC transitions, is still relatively high.

With a further increase in temperature, the frequency of *ν*_s_(CH_2_) increases to 2851.5 cm^−1^ at *T* = 53.0 °C and finally to 2852.0 cm^−1^ at *T* = 70.3 °C. This increase is again attributed to an increased amount of *gauche* conformers and a higher flexibility of the alkyl chains. The corresponding cooling curve shows the same pattern except for a hysteresis of 5–7 K, which is indicative for a hindered reformation of the ordered fibrous aggregates. A comparable hysteresis is visible in the DSC scan.

#### TEM

**Figure 3:**
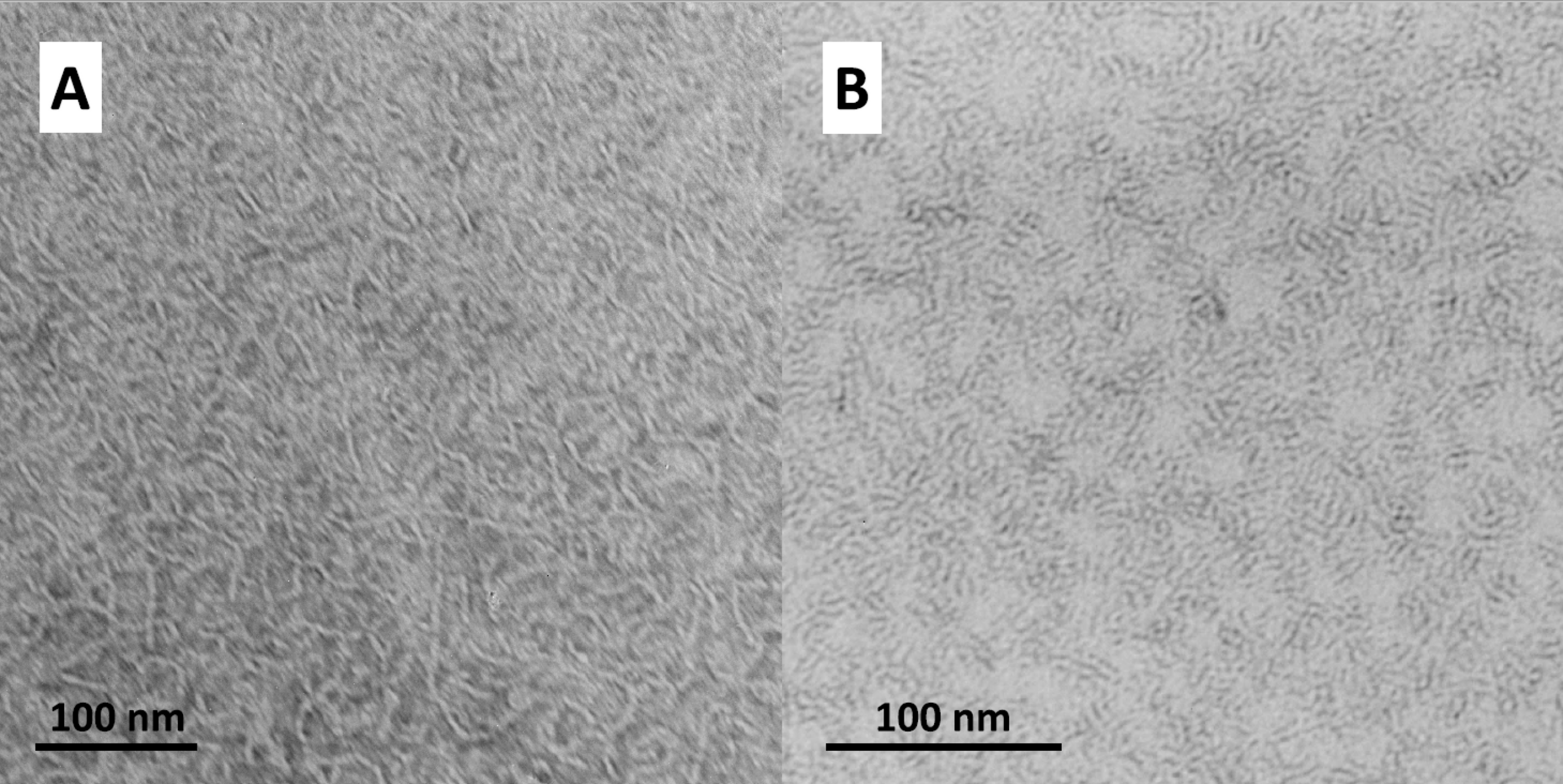
TEM image of an aqueous suspension (*c* = 0.05 mg mL^−1^) of PC-C18pPhC18-PC. The samples were prepared at about 7 °C (A) or at 36 °C (B) and stained with uranyl acetate before drying.

To analyse the structure of aggregates, samples for TEM were prepared below the first transition as well as between the first and the second transition of PC-C18pPhC18-PC. At about 7 °C, the TEM image depicts a dense network of long nanofibres (A). The morphology of these fibres changes with increasing temperature. At 36 °C, the TEM image shows the presence of flexible nanofibres; no small micellar aggregates were found (B) (Figure 3). This means, the first endothermic transition of PC-C18pPhC18-PC is connected to a fibre–fibre transformation and not to a fibre–micelle transformation as found for PC-C17pPhC17-PC [35] and also for unmodified, phenylene-free bolalipids PC-C*n*-PC with alkyl chain lengths (*n*) up to 32 carbon atoms [24,38]. But, bolalipids with very long alkyl chain, namely PC-C34-PC and PC-C36-PC, show an additional fibre–fibre transition, where the flexibility of bolalipid molecules is slightly increased. However, due to the very long alkyl chains, the remaining van-der-Waals contacts are sufficient for the formation of fibrous aggregates above the first transition [26,40]. This seems to be also the case for PC-C18pPhC18-PC.

#### SANS

**Figure 4:**
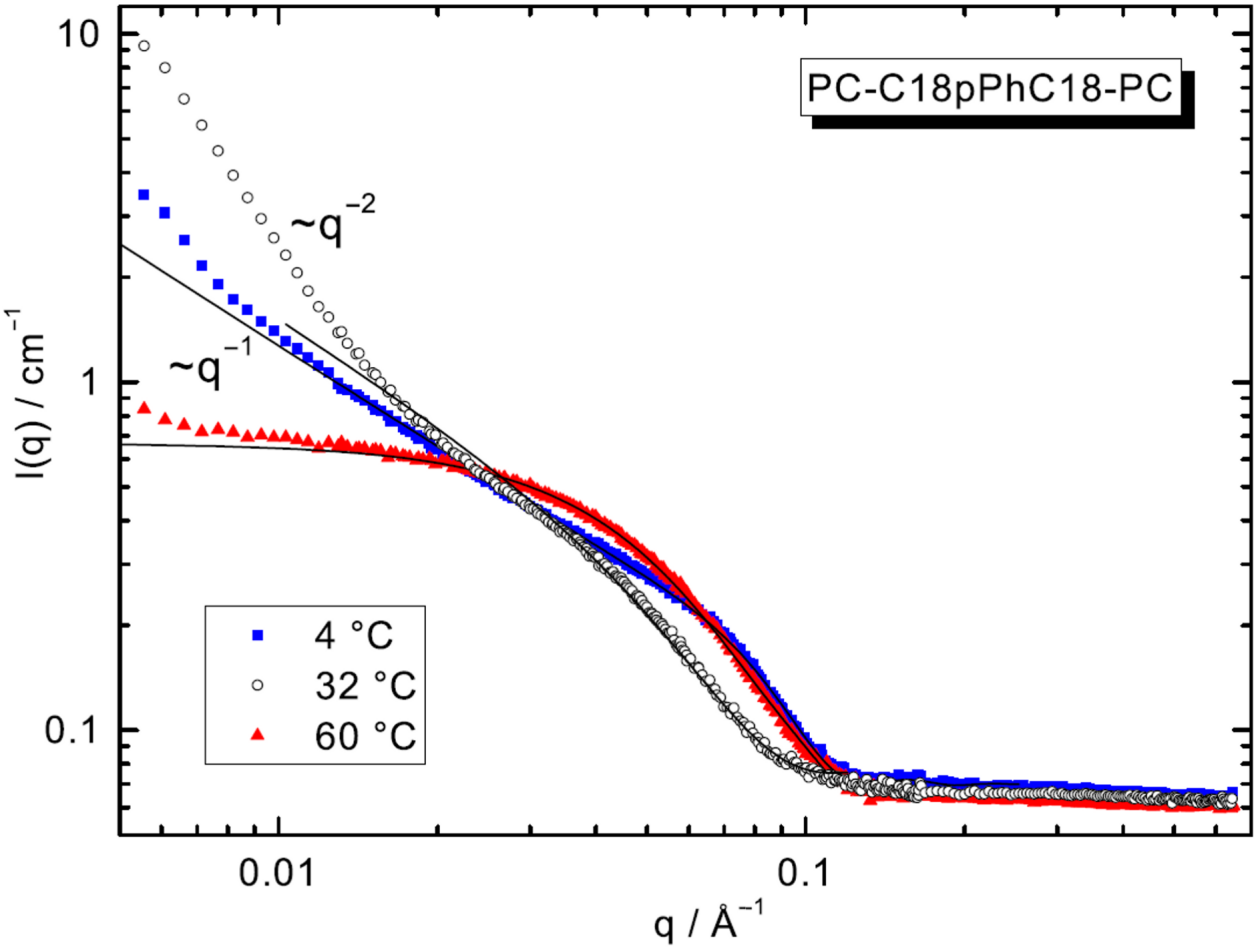
SANS data of a suspension of PC-C18pPhC18-PC (*c* = 1 mg mL^−1^ in D_2_O, scattered data) with IFT analysis (solid lines) at different temperatures.

In order to ensure the results obtained with TEM and IR, SANS measurements were performed (*c* = 1 mg mL^−1^ in D_2_O). SANS data follows the structural changes of bolalipid aggregates with varying temperature and shows firstly an increase of scattering intensities with temperature change from 4 °C to 32 °C, accompanied with an increase of slope (α) of scattering intensities approximated by power law, i.e., *I*(*q*) ~ *q^−^*^α^ at lower *q*-range from 1 to 2 (Figure 4). A further increase in temperature to 60 °C leads to a decrease of scattering intensities and a decrease of α almost to zero. The observation of slope α equals −1 is a signature of scattering from elongated and rigid objects of length *L* and radius *R* in an interval of scattering vectors 1/*L* < *q* < 1/*R*. Scattering data have been analysed via indirect Fourier transformation (IFT) method (Figure 4, solid lines) for infinitely long cylinder described previously [43–46] and the cross section pair distance distribution function, *p_CS_(r)*, has been obtained (Supporting Information File 1, Figure S1). This distribution function gives values of the diameter of cross section of ≈ 50–55 Å and an aggregation number *N_agg_* of about 9 ± 1 bolalipid molecules per 1 nm length of cylinder at *T* = 4 °C.

At 32 °C – a temperature between the first transition and the second transition of PC-C18pPhC18-PC – SANS intensities are proportional to *q*^−2^ and, hence, suggest the scattering from flexible chains. Here, the *q*-value measured is *L*_cont_^−1^ < *q* < *l_p_*^−1^, where *l_p_* is the persistence length of flexible aggregates and *L_cont_* is the contour length of the aggregate. The crossover between the region of rigid cylinder, i.e., the plateau of Holtzer [47] *qI*(*q*) versus *q* (Supporting Information File 1, Figure S3), to increase at lowest *q* (flexibility region) takes place around *q* = 0.02 Å^−1^, which suggests that aggregates become flexible in length scale above 100 Å. It can be concluded that an increase of temperature from 4 °C to 32 °C shifts system from long and rigid fibres to flexible chains. Additionally, also the cross-sectional parameters of flexible chain changes, i.e., the diameter increases up to 70 Å (Supporting Information File 1, Figure S1) and *N*_agg_ slightly increases to 10 ± 1 bolalipid molecules per 1 nm length of flexible chain.

At 60 °C, the scattering intensities at lowest *q*-range are typical for scattering from small globular objects such as micelles. Mean diameter *D* of these objects (*q* << 1/*D*) and *p*(r) has been obtained in approximation of 3D-objects, where all three axes of the object have the same order of magnitude. At 60 °C bolalipid molecules form small, elliptical-like micelles of maximal diameter of 200 Å (Supporting Information File 1, Figure S2). The radius of gyration (*R*_g_) is around 32 ± 2 Å, which equals a diameter of 83 Å, and *N*_agg_ is about 121 ± 5 bolalipid molecules per micelle. Modelling of the scattering curves by ellipsoids of revolution yields values for the semi axes *a* = *b* = 22 Å and *c* = 47 Å. The results of SANS measurements are summarized in Table 1.

**Table 1:** SANS data obtained from IFT analysis for aqueous suspensions of PC-C18pPhC18-PC in D_2_O at different temperatures.^a^

*T* [°C]	aggregate shape	*D_max_* [Å]	*I*(0) [cm g^−1^] or *I*_CS_(0) [Å^−1^ cm g^−1]^	*M* [g] or *M*_L_ [g cm^−1]^	*N_agg_* or *N_agg_* [nm^−1]^	*R_g_* [Å] or *R*_CS,g_ [Å]	*R* [Å]	*a* [Å]	*b* [Å]	*L* [Å] or *c* [Å]

4	fibres (stiff cylinders)	45	3.96 ± 0.01	1.37 × 10^−13^	8.7 ± 1	16.40 ± 0.02	23.2	22 ± 2	22 ± 2	>2000
32	flexible fibres	70	4.61 ± 0.01	1.59 × 10^−13^	10 ± 1	24.25 ± 0.03	34.3	30 ± 3	30 ± 3	>2000

60	micelles	100	590 ± 10	1.91 × 10^−19^	121 ± 5	32 ± 2	41.3	22 ± 2	22 ± 2	47 ± 4

^a^*D*_max_: maximal size or cross section of aggregate; *I*(0): scattering at “zero angle”; *I*_CS_(0): scattering at “zero angle” of cylindrical cross section; *M*: mass; *M*_L_: mass per unit length; *N*_agg_: aggregation number of micelles or number of molecules per unit length of rod-like object; *R*_g_: radius of gyration; *R*_CS,g_: radius of gyration of cross-section; *R* and *R*_CS_ are effective radius of aggregate or radius of cylindrical cross section in homogeneous approximation; *a*, *b*, *c*, and *L*: values for semi axes *a* and *b*, and estimated length *L* for fibres with circular cross-section and for semi axes of ellipsoid of revolution of micelles *a* = *b*, and *c*.

### Mixing behaviour with saturated phosphatidylcholines

In order to investigate the miscibility of our new bolalipid with bilayer-forming phospholipids, PC-C18pPhC18-PC was mixed with the double-chain phospholipids DMPC, DPPC, and DSPC, i.e., phosphatidylcholines with saturated alkyl chains of different length. In a previous study we showed that unmodified bolalipids, such as PC-C32-PC, could not be incorporated into bilayers of DMPC or POPC [28]. In contrast, bolalipids with an alkyl chain modification, e.g*.*, PC-C17pPhC17-PC, were partially miscible with DPPC and DSPC. But, closed lipid vesicles (liposomes) and a pronounced stabilization of the lamellar structure – prerequisites for the use as drug delivery vehicle – could not be observed, and bilayer fragments as well as elongated micelles were formed instead [35]. The isomers PC-C17mPhC17-PC and PC-C17oPhC17-PC, bearing a *meta* or *ortho* substitution at the central phenyl ring, showed the formation of disk-like aggregates in mixtures with DPPC and DSPC, respectively, with partly increased thermal stability [34]. The mixing behaviour of PC-C18pPhC18-PC with phosphatidylcholines was now studied by means of DSC and TEM. Additionally, one example of a bolalipid/phospholipid mixture was further investigated by FTIR and SAXS measurements.

#### DSC

**Figure 5:**
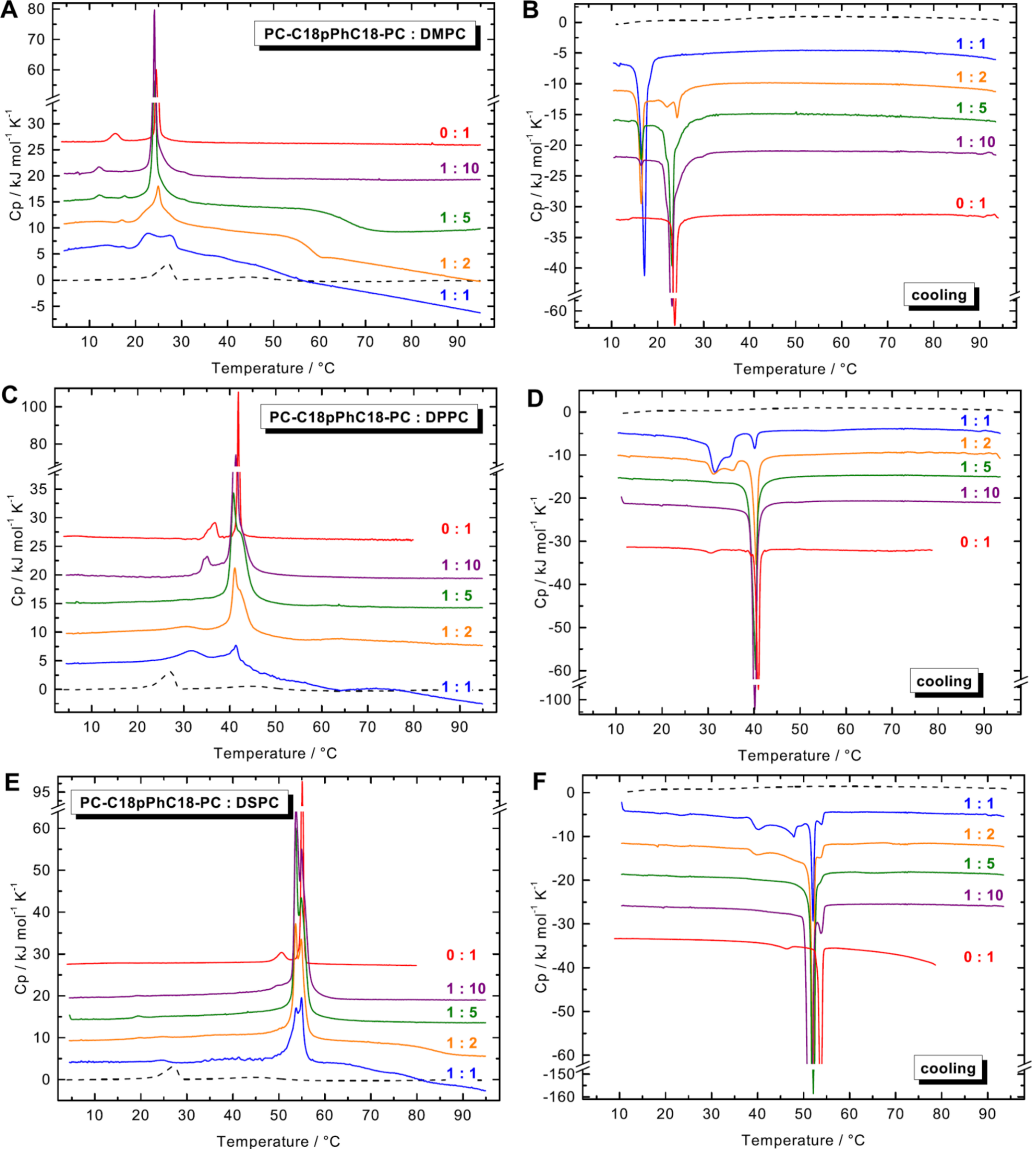
DSC heating (left-hand column) and cooling (right-hand column) scans of different PC-C18pPhC18-PC:phospholipid mixtures (*c* = 3 mM in phosphate buffer, pH 7.6): DMPC (A,B), DPPC (C,D), and DSPC (E,F). The molar ratios are displayed in the graph. The DSC data of pure PC-C18pPhC18-PC (black dashed line, *c* = 1 mg mL^−1^) and pure phospholipids (red solid line, *c* = 1 mg mL^−1^) are shown for comparison. The heating and cooling rate was 60 K h^−1^. The curves are shifted vertically for clarity.

We firstly investigated the thermotropic behaviour of aqueous suspensions of PC-C18pPhC18-PC:phospholipid mixtures (*c* = 3 mM) with different molar ratios, namely 1:10, 1:5, 1:2, and 1:1 (*n*:*n*). The heating scans are depicted in the left-hand column of Figure 5, the corresponding cooling scans are shown in the right-hand column of Figure 5.

The DSC heating scans of the pure, bilayer-forming phospholipids show the two well-known endothermic transitions (see red solid lines in Figure 5): the pre-transition from the L_β’_-phase to the ripple-phase (P_β’_) and the very cooperative main transition to the L_α_-phase, where the alkyl chains are fluidized due to an increased amount of *gauche* conformers.

The DSC heating scan of a 1:10 mixture of PC-C18pPhC18-PC and DMPC (Figure 5A) shows a sharp peak at *T* = 24.1 °C, below *T*_m_ of the pure DMPC. With increasing amount of bolalipid, this peak stays nearly at the same temperature but gets broader. In the equimolar mixture of PC-C18pPhC18-PC and DMPC, the DSC scan shows a broad transition with two peaks at *T* = 22.8 °C and *T* = 27.3 °C. The latter one corresponds to *T*_m_ of the pure bolalipid. In addition, one or two very small pre-transitions are detectable below the main transition. In the corresponding DSC cooling scans (Figure 5B), a biphasic transition appears in most mixtures. Starting with the 1:10 mixture, the DSC scan depicts a peak at *T* = 23.1 °C, slightly below *T*_m_ of pure DMPC. With increasing amount of the bolalipid, this peak disappeared gradually and at the same time a second peak at *T* = 16.5 °C emerged, which shifts to *T* = 17.1 °C in the 1:1 mixture.

In the mixtures with DPPC (Figure 5C), the DSC heating scan of the 1:10 ratio shows an endothermic transition at *T* = 41.4 °C, 0.5 K below the *T*_m_ of pure DPPC, including a high-temperature shoulder. With increasing amount of bolalipid, this peak disappears gradually. In the 1:2 mixture and more pronounced in the 1:1 mixture, a second, broad peak emerged at *T* ≈ 32 °C, which is above *T*_m_ of the pure bolalipid. The corresponding DSC cooling scans (Figure 5D) depicts a sharp transition peak at around 40.2 °C, again slightly below *T*_m_ of pure DPPC, which decreases gradually with increasing amount of bolalipid. In the 1:2 and 1:1 mixture, two additional low-temperature peaks appear at 34.7 °C and 31.5 °C, indicating a complex behaviour.

A comparable situation is found for mixtures of PC-C18pPhC18-PC with DSPC. Here, the DSC heating scan of the 1:10 mixture (bolalipid:phospholipid) shows a main transition with a splitting (53.7 °C and 54.9 °C; Figure 5E), again slightly below *T*_m_ of pure phospholipid. With increasing amount of bolalipid, both transition peaks decrease in intensity but virtually stay at the same temperature. The DSC cooling scan (Figure 5F) of the 1:10 mixture shows a peak at about 52 °C. With increasing amount of PC-C18pPhC18-PC, this peak stays at the same temperature and decreases gradually. Finally, the cooling scan of the 1:1 mixture shows again several peaks: the most distinct ones at 52.1 °C, and three smaller ones at 53.9 °C, 47.8 °C, and 40.2 °C.

In the DSC heating curve of some of our bolalipid/phospholipid mixtures, an additional exothermic peak as well as a continuous or stepwise decrease in the heat capacity is observed during heating. It is conceivable that a metastable state is reached after *T*_m_. This kinetically stable state transforms within the timescale of the DSC experiment (heating rate = 60 K h^−1^) into a thermodynamically stable state, which is accompanied by an exothermic heat effect showing up as broad transition peak or as decrease in *Cp*. The notion of two different states is supported by the fact that this exothermic peak or the decrease in *Cp* disappeared using a slower heating rate of 20 K h^−1^ (data not shown). A similar effect was found previously for mixtures of PC-C17pPhC17-PC [35] and PC-C32-10,10’Me-PC, a bolalipid with two methyl groups within the long alkyl chain [48], with different saturated phosphatidylcholines.

#### TEM

**Figure 6:**
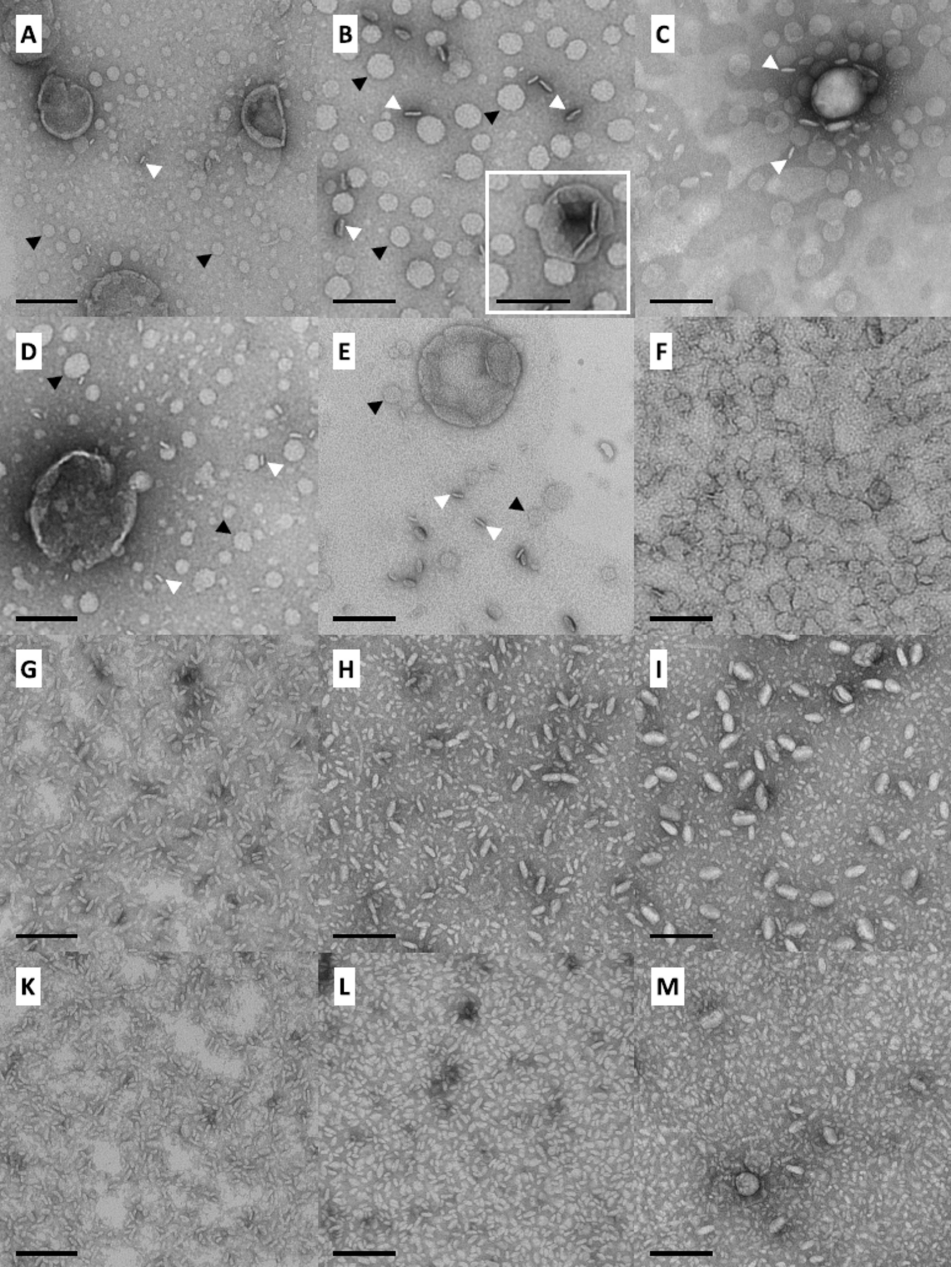
TEM images of aqueous suspensions (*c* = 60 µM or *c* = 30 µM for C) of PC-C18pPhC18-PC/phospholipid mixtures with DMPC (left-hand column), DPPC (middle column), or DSPC (right-hand column) in different bolalipid/phospholipid ratios: 1:10 (A–C), 1:5 (D–F), 1:2 (G–I), and 1:1 (K–M). Samples were prepared at 22 °C and stained with uranyl acetate before drying. The bar corresponds to 100 nm.

To get an idea about the aggregate structure of the mixed systems in aqueous suspension TEM images were recorded from negatively stained samples. All specimens were prepared at about 22 °C, i.e., in the case of mixtures with DMPC at the beginning of the main transition and for mixtures with DPPC and DSPC, respectively, below the transitions observed in DSC. The images are shown in Figure 6. By comparing the images of the different phospholipid mixtures (DMPC, DPPC or DSPC) with same mixing ratio, one can see that the shape of aggregates is similar.

Starting with the lowest bolalipid content, EM images of the 1:10 mixture reveal the presence of crashed vesicles as well as disk-like aggregates (Figure 6A–C). The disks, which are particularly found in mixtures with DPPC, are nearly round shaped and they are oriented either parallel (black arrow head) or perpendicular to the grid surface (white arrow head). EM images of a bolalipid/phospholipid = 1:5 mixture show the existence of disk-like aggregates of comparable size for all three different phospholipids (Figure 6D–F). However, also some collapsed vesicles are present in the mixture with DMPC and DPPC. By increasing the bolalipid content to a 1:2 mixture (bolalipid/phospholipid), the shape of aggregates changes to small, elongated micelles (Figure 6G–I). In the case of DPPC and DSPC mixtures, another kind of larger aggregates is formed. Lastly, for the equimolar mixtures (Figure 6K–M), EM images show again the formation of small, elongated micelles. Only in the case of the DSPC mixture, some larger aggregates are found.

We conclude that PC-C18pPhC18-PC is not completely immiscible with bilayer-forming phosphatidylcholines and that the PC-C18pPhC18-PC is partially miscible with these phospholipids. When ca. 10 or 20 mol % of bolalipid is inserted in membranes of phosphatidylcholines, disk-like structures are the dominant aggregate form. We can only speculate, whether both types of lipid molecules are randomly distributed within the disks or not. It is also conceivable that a partial demixing occurs inside the disk and that bolalipid molecules are accumulated at the rim of this disk stabilizing them against fusion into larger aggregates. With increasing amount of bolalipid, micellar structures became dominant. It seems conceivable that, in the case of 1:1 and 1:2 mixtures, either both components are not homogeneously distributed within the aggregates or two different species of aggregates exists, bolalipid-rich ones and phospholipid-rich ones (see for example Figure 6H or I, showing two different sizes of micelles). This is in accordance with the DSC measurements shown above, where a very broad peak or two peaks are observed in the mixture of, e.g., PC-C18pPhC18-PC and DMPC (Figure 5A) or DPPC (Figure 5C).

Similar disk-like assemblies (bicelles) were also reported for phospholipid mixtures, for example in DPPC/DHPC systems [49], or for mixtures of phospholipids with other amphiphilic substances, e.g., in PEG-stabilized bilayer systems [50–51], for phospholipids with membrane scaffold proteins [52–53] or in combination with copolymers [54–55], or for DPPC in mixture with a T-shaped amphiphile [56].

#### FTIR spectroscopy and SAXS of PC-C18pPhC18-PC/DPPC (1:1)

**Figure 7:**
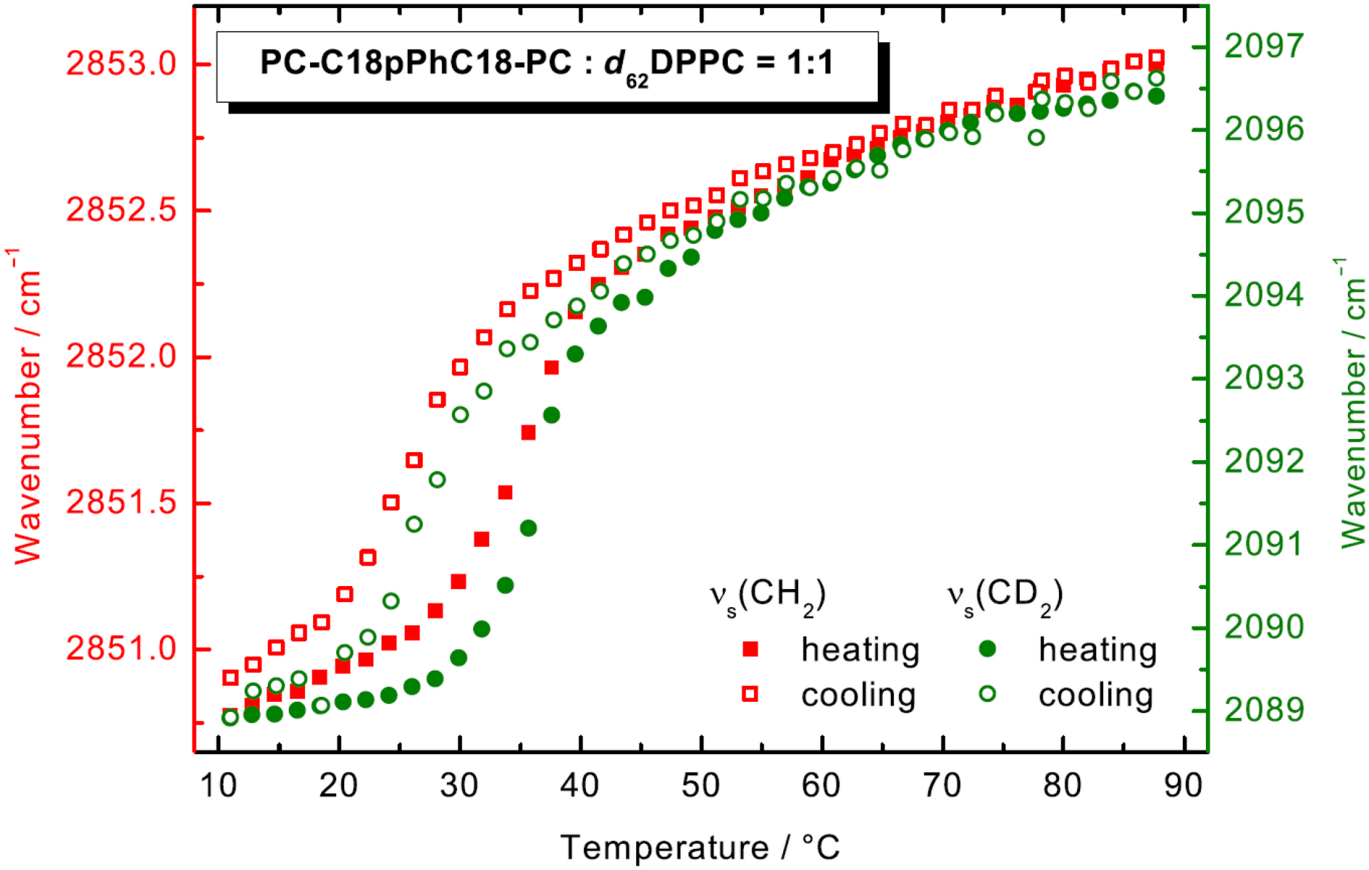
FTIR spectroscopic data (symmetric CH_2_ stretching vibration: red squares, right axis; symmetric CD_2_ stretching vibration: green circles, left axis) of an equimolar mixture of PC-C18pPhC18-PC:d_62_-DPPC (*c* = 50 mg mL^−1^ in phosphate buffer, pH 7.6, heating: filled symbols, cooling: open symbols).

To support the finding of disk-like aggregates seen by TEM, one mixture was exemplarily analyzed by means of FTIR and SAXS. Temperature dependent FTIR measurements were used to clarify the question whether the transition of both PC-C18pPhC18-PC and DPPC happen simultaneously. To distinguish between both components, DPPC with fully deuterated alkyl chains (*d*_62_-DPPC) was used and the change in the position of the stretching vibrational band of CH_2_ (from the bolalipid; Figure 7 red symbols) and CD_2_ (from the phospholipid; Figure 7 green symbols) was followed. The deuteration of alkyl chains in phospholipids usually causes a shift of the main transition by about 5 K to lower temperatures, as shown by Petersen et al. [57].

At *T* = 10.9 °C, the frequency of *ν*_s_(CH_2_) is at 2850.8 cm^−1^, which is comparable to the frequency of *ν*_s_(CH_2_) of the pure PC-C18pPhC18-PC at *T* = 30 °C, above the first transition in DSC (see above). Hence, the alkyl chains of the bolalipid contain a small amount of *gauche* conformers. The frequency of *ν*_s_(CD_2_) is at 2088.9 cm^−1^ at *T* = 10.9 °C, indicating ordered alkyl chains in all-*trans* conformation for the deuterated phospholipid. With increasing temperature, the frequencies of both band increase with a distinct jump to 2852.3 cm^−1^ and 2093.9 cm^−1^ at 43.4 °C and further to 2853.0 cm^−1^ and 2096.4 cm^−1^ at 87.7 °C. This increase is attributed to an increased amount of *gauche* conformers within both deuterated and non-deuterated alkyl chains. However, the transition from the gel phase to the liquid-crystalline phase seems to be slightly different for both components and did not occur simultaneously. The increase in frequency for *ν*_s_(CH_2_) (bolalipid) starts at lower temperature than for *ν*_s_(CD_2_) (phospholipid), which stays nearly constant up to 30 °C. One explanation for this behaviour could be that at low temperatures, the phospholipid forms bilayer fragments that are stabilized by bolalipid molecules, possibly in a U-shaped conformation, at the rim of the disk.

**Figure 8:**
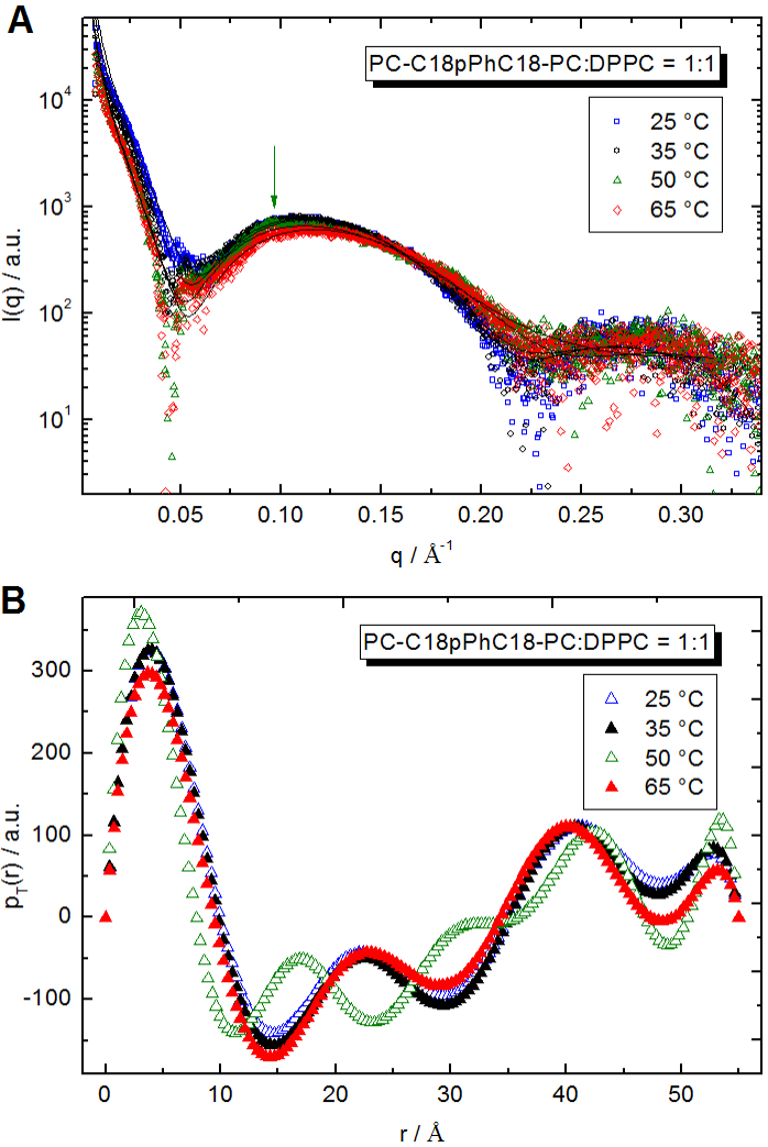
(A) SAXS diffractograms of an equimolar mixture of PC-C18pPhC18-PC/DPPC (*c* = 10 mg mL^−1^, scattered data) in phosphate buffer (pH 7.6) at different temperatures and IFT fits (solid lines). The arrow points to diffraction maximum (at *T* = 50 °C) of multilayer object. (B) Pair distance distribution function of thickness of discs obtained at different temperatures of an equimolar mixture of PC-C18pPhC18-PC/DPPC (*c* = 10 mg mL^−1^) in phosphate buffer (pH 7.6).

To analyze the aggregates in more detail, SAXS measurements at different temperatures were performed (Figure 8A). Scattering data do not show sharp diffraction maxima, which are observed for binary DPPC/water mixtures at similar temperature and concentration range [34]. The transformation from sharp maxima to broad ones points on structural changes of the lipid system from multilamellar objects for pure DPPC to separated lamellae for the PC-C18pPhC18-PC:DPPC mixtures. Only at *T* = 50 °C, a very small diffraction peak has been observed (see arrow in Figure 8A), which corresponds to a lamellar repeat distance (bilayer thickness plus an interlamellar water layer) of about 67 Å.

Firstly, the slope at low *q*-range has been determined as −2, which corresponds to the scattering from flat objects, i.e., aggregates, where the size of two dimensions is much larger than the third dimension. In order to get structural information such as a distribution of scattering length density along the smaller dimension, IFT analysis in approximation of infinitely thin objects has been applied [45–46]. The thickness pair distance distribution function *p_T_*(*r*) describes the scattering intensity via:

[1]
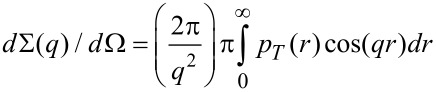


The *p_T_(r)* function is connected with the scattering contrast between object and solvent and is written as

[2]
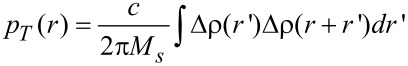


where *r* is the coordinate in the direction normal to the surface of the disk-like objects and *M*_S_ is connected with mass per surface unit. IFT satisfactorily describes our experimental data (solid lines in Figure 8A). The *p_T_*(*r*) functions for the equimolar PC-C18pPhC18-PC/DPPC mixtures versus temperature are shown in Figure 8B. The negative values of *p_T_*(*r)* in the intermediate *r*-range could be explained by different sign of the scattering contrast for alkyl chains (positive) and polar groups (negative) of DPPC in water.

IFT analysis requires as input parameter the maximal size of objects, whose numerical solution is searched for. In present analysis, the maximal thickness of disks is needed. To get a sufficient fit of experimental data and a stable shape of *p_T_*(*r*) function, a maximal thickness of 55 Å has been applied. The comparison with pure DPPC bilayers (without additives) confirms that an addition of PC-C18pPhC18-PC does not change the thickness of the DPPC bilayer [58]. Also, a significant decrease of bilayer thickness with increasing temperature was not observed; only a small change for large *r* (region of polar groups) for 65 °C has been detected. The difference found for 50 °C can be connected that in these conditions also some multilamellar objects are formed.

## Conclusion

The PC-C18pPhC18-PC, a long-chain, phenylene-modified bolalipid, self-assembles at room temperature into nanofibres, which leads to a gelation of the suspension. With increasing temperature, the stiff fibres transform into more flexible ones. This transformation is accompanied with an endothermic transition observed in DSC measurements. The existence of fibrous aggregates after the first DSC transition peak is remarkable since the analogue bolalipid, PC-C17pPhC17-PC with a slightly shorter alkyl chain, shows a direct transformation from nanofibres into small micelles. For PC-C18pPhC18-PC, small micellar aggregates are only observed after the second transition observed by DSC. Hence, the elongation of the alkyl chain for two methylene units is sufficient to stabilize the flexible fibres above the first transition via van-der-Waals interactions.

The mixing of PC-C18pPhC18-PC with bilayer-forming phosphatidylcholines (DMPC, DPPC, or DSPC) leads in most cases to the formation of small elongated micelles, bilayer fragments, or disk-like aggregates. However, an increased thermal stability of these aggregates could not be observed. For PC-C18pPhC18-PC:DPPC mixtures with an excess of phospholipid, the formation of virtually uniform, disk-like aggregates is observed. The arrangement of both lipid components inside these disks is not fully understood, but it seems very likely that a partial demixing occurs and that the rims of the bilayer disks are stabilized by PC-C18pPhC18-PC molecules in a U-shaped conformation.

## Experimental

### Substances

DMPC and DPPC were obtained from Lipoid KG (Ludwigshafen, Germany). DSPC was purchased from Sygena AG (Switzerland). *d*_62_-DPPC was obtained from Avanti Polar Lipids (Alabaster, AL, USA). The synthetic procedure and the analytical data of the newly prepared bolalipid PC-C18pPhC18-PC are described in detail in Supporting Information File 1.

### Methods

#### Sample preparation

In a similar manner to a procedure from [34], the appropriate amount of the bolalipid PC-C18pPhC18-PC was suspended in H_2_O (Milli-Q). Homogeneous suspensions were obtained by heating to 90 °C and vortexing. Binary lipid mixtures were prepared from lipid stock solutions in CHCl_3_/MeOH (2/1, v/v) as solvent by mixing appropriate volumes of the stock solutions. The organic solvent was then removed in a stream of N_2_. The resulting lipid films were kept in an evacuated flask for 24 h to remove residual traces of solvent. The suspensions were then prepared by adding a certain volume of aqueous phosphate buffer (10 mM, pH 7.6) to obtain a total lipid concentration of 3 mM. The samples were vigorously vortexed at 60 °C to obtain a homogeneous suspension.

#### Transmission electron microscopy (TEM)

The samples were prepared by spreading 5 µL of the bolalipid suspension (*c* = 0.05 mg mL^−1^ in case of pure bolalipid, *c* = 60 µM in case of lipid mixtures) onto a copper grid coated with a Formvar film. After 1 min, excess liquid was blotted off with filter paper and 5 µL of 1% aqueous uranyl acetate solution were placed onto the grid and drained off after 1 min. Specimens prepared below ambient temperature (*T* ≈ 7 °C) were dried for 2 days at this temperature and kept in an desiccator at ambient temperature. Specimens, which were prepared in a modified drying oven above ambient temperature, were further dried for 1 h at the appropriate temperature and finally kept in an desiccator at room temperature. All specimens were examined with a Zeiss EM 900 transmission electron microscope (Carl Zeiss Microscopy GmbH, Jena, Germany).

#### Differential scanning calorimetry (DSC)

DSC measurements were performed using a MicroCal VP-DSC differential scanning calorimeter (MicroCal Inc. Northampton, MA, USA). Before the measurements, the sample suspension and the water (or phosphate buffer) used as a reference were degassed under vacuum while stirring. A heating rate of 60 K h^−1^ was used, and the measurements were performed in the temperature interval from 5 °C to 95 °C. To check the reproducibility, three consecutive scans were recorded for each sample. The water–water and buffer–buffer baseline, respectively, was subtracted from the thermogram of the sample, and the DSC scans were evaluated using MicroCal Origin 8.0 software.

#### Fourier-transform infrared spectroscopy (FTIR)

Infrared spectra were collected using a Bruker Vector 22 Fourier transform spectrometer with DTGS detector operating at 2 cm^−1^ resolution. The bolalipid suspension (PC-C18pPhC18-PC: *c* = 50 mg mL^−1^ in H_2_O; PC-C18pPhC18-PC/*d*_62_-DPPC, 1:1, *n*/*n*, *c* = 100 mg mL^−1^ in phosphate buffer 300 mM, pH 7.7) was placed between two CaF_2_ windows, separated by a 6 µm spacer. IR spectra were recorded in steps of 2 K in the temperature range 9 °C to 71 °C or 9 °C to 89 °C. After an equilibration time of 8 min, 64 scans were recorded and accumulated. The corresponding spectra of the solvent (H_2_O or D_2_O) were subtracted from the sample spectra using the OPUS software supplied by Bruker.

#### Small angle neutron scattering (SANS)

SANS measurements were made on the steady state reactor based pin-hole SANS instrument Quokka [59] which is found on a cold guide at the Australian Nuclear Science and Technology Organization’s (ANSTO) research reactor OPAL (Lucas Heights, Australia). Sample (PC-C18pPhC18-PC, *c* = 1 mg mL^−1^ in D_2_O) was placed in cylindrical quartz cuvettes of path length 2 mm. SANS spectra were recorded on a position sensitive detector consisting of 192 × 192 pixels (5 × 5 mm²) at 3 sample-to-detector distance 1.3, 6, and 12 m using neutrons of wavelength, λ = 5.0 Å (Δλ/λ = 10%). After correcting the raw data for the sensitivity of each detector pixel, masking the beam stop shadow, subtracting backgrounds consisting of the sample buffer in identical samples cells and normalizing to the empty beam intensity the radially averaged isotropic scattering data from each sample-to-detector distance were joined to produce a continuous *q*-range of 0.009 to 0.7 Å^−1^, where *q =* 4π × sin(2θ)/λ and θ is the scattering angle. This was achieved using macros modified for the program IgorPro (version 6.34, WaveMetrics, Inc. 2013) from those macros written for the NIST Center for Neutron Research (Gaitherburg, USA) SANS instruments [60].

#### Small angle X-ray scattering (SAXS)

SAXS experiments were performed at the P12 BioSAXS beamline of the European Molecular Biology Laboratory (EMBL) at the storage ring PETRA III of the Deutsche Elektronen Synchrotron (DESY, Hamburg, Germany) using a Pilatus 2M detector (1475 × 1679 pixels; Dectris, Switzerland) and synchrotron radiation with a wavelength λ = 1 Å. The sample-to-detector distance was 4 m, allowing for measurements in the *q*-range interval from 0.75 Å^−1^ to 5 Å^−1^. The *q*-range was calibrated using the diffraction patterns of silver behenate. The experimental data were normalized to the incident beam intensity, corrected for non-homogeneous detector response, and the background scattering of the aqueous buffer was subtracted. Sample (PC-C18pPhC18-PC/DPPC, 1:1, *n*/*n*, *c* = 10 mg mL^−1^ in phosphate buffer pH 7.4) has been placed in 1 mm glass capillaries. Temperature has been controlled by Linkam heating stage HFSX 350 (Surrey, UK) with accuracy ± 0.1 °C. Twenty consecutive frames (each 0.05 s) comprising the measurement of the solvent (phosphate buffer pH 7.4) and sample were performed. In order to verify that no artefacts as a result of radiation damage occurred, all scattering curves of a recorded dataset were compared to a reference measurement (typically the first exposure) and finally integrated by automated acquisition program given by Franke et al. [61].

## Supporting Information

File 1 Experimental procedures, characterization data for synthesized compounds and further SANS data.
