# Supplementary material for: Aggregation behaviour of a single-chain, phenylene-modified bolalipid and its miscibility with classical phospholipids
Source: Beilstein J Org Chem. 2017 May 23;13:995–1007. doi: 10.3762/bjoc.13.99 (PMC5480355; doi:10.3762/bjoc.13.99)
Supplement: File 1 — Experimental procedures, characterization data for synthesized compounds and further SANS data. [file Beilstein_J_Org_Chem-13-995-s001.pdf]

Supporting Information

for

Aggregation behaviour of a single-chain, phenylene-modified bolalipid and its miscibility with classical phospholipids

Simon Drescher[*,1], Vasil M. Garamus[2], Christopher J. Garvey[3], Annette Meister[4,5] and Alfred Blume[4]

Address: [1]Institute of Pharmacy, Martin Luther University (MLU) Halle-Wittenberg, Wolfgang-Langenbeck-Str. 4, 06120 Halle (Saale), Germany, [2]Helmholtz-Zentrum Geesthacht (HZG), Centre for Materials and Costal Research, Max-Planck-Str. 1, 21502 Geesthacht, Germany, [3]Australian Nuclear Science and Technology Organisation (ANSTO), Kirrawee DC, NSW, Australia, [4]Institute of Chemistry, MLU Halle-Wittenberg, von-Danckelmann-Platz 4, 06120 Halle (Saale), Germany and [5]Institute of Biochemistry and Biotechnology, MLU Halle-Wittenberg, Kurt-Mothes-Str. 3, 06120 Halle (Saale), Germany.

Email: Simon Drescher - simon.drescher@pharmazie.uni-halle.de

*Corresponding author

Dedicated to Prof. Dr. Bodo Dobner on the occasion of his 65th birthday.

Experimental procedures, characterization data for synthesized compounds and further SANS data.

Content:

1. Syntheses S2
1.1 Methods S2
1.2 Synthetic procedures and analytical data of compounds S3
2. Physicochemical investigations S5
2.1 SANS S5
3. References S7

1. Syntheses

1.1 Methods

Chemicals for the synthesis were purchased from Sigma Aldrich Co. (Steinheim, Germany) and were used without further purification. 2-Bromoethylphosphoric acid dichloride was prepared according to the literature [1]. All solvents for synthetic purposes were dried and distilled before use. The purity of all compounds was checked by thin-layer chromatography (TLC) using silica gel 60 F254 plates (Merck, Darmstadt, Germany). The chromatograms were developed by means of bromothymol blue. Purification of the diol was carried out by recrystallization or by middle pressure liquid chromatography (MPLC; *Büchi*, Essen, Germany) on silica gel (0.040–0.063 mm, *Merck*). The MPLC was equipped with a Fraction Collector C-660, Pump Module C-601 (2×), Pump Manager C-615 and UV detector (cut off = 254 nm), and the following solvent was used for elution: 25 min isocratic chloroform, 60 min continuous increase from chloroform/diethyl ether (100/0, v/v) to (60/40, v/v); flow = 20 mL/min, sample fractions of 20 mL. The purification of the final bolalipid was carried out using chromatography and chloroform/methanol/water as eluent and the gradient technique.

Melting points were determined with Boetius apparatus. Elemental analyses (C, H, N) were conducted using a Leco CHNS-932 (Leco-Corporation, St. Joseph, USA). ^{1}H and ^{13}C NMR spectra were recorded on a Varian Gemini 2000 spectrometer or a Varian Inova 500 with the use of $CDCl_3$ or CD_3OD as internal standard. Chemical shifts (δ) are reported in parts per million (ppm). The coupling constants (*J*) are reported in Hz. Mass spectrometric (MS) data were obtained with a Finnigan LCQ-Classic (ESI–MS) (Thermo Seperation Products, San José, USA) or were recorded on an AMD 402 (70 eV) spectrometer (EI–MS) (AMD Intecta GmbH, Harpstedt, Germany). High-resolution mass spectra (HRMS) were recorded on a Thermo Fisher Scientific LTQ-Orbitrap mass spectrometer with static nano-electrospray ionization (Thermo Fisher Scientific Inc., Waltham, USA).

1.2 Synthetic procedures and analytical data of compounds

Bis-Sonogashira cross-coupling: According to a procedure described previously [2], an oven-dried flask was filled with 1,4-dibromobenzene (190 mg, 0.8 mmol), octadec-17-yn-1-ol (Ac-C16-OH) [3-4] (506 mg, 1.9 mmol), $PdCl_2(PPh_3)_2$ (33 mg, 6 mol %) and TBAF×$3H_2O$ (950mg, 3 mmol) under argon atmosphere. The mixture was subsequently stirred at 80 °C for 1–2 h until complete consumption of the starting material. Afterwards, 30 mL H_2O was added and the mixture was extracted with chloroform (3 × 20 mL). The combined organic layers were washed with brine (20 mL), water (20 mL), dried over sodium sulfate, and evaporated. The residue was purified by MPLC using chloroform/diethyl ether as eluents and gradient technique to afford the 18,18'-(1,4-phenylene)bis(octadec-17-yn-1-ol) (HO-C16pAcPhAcC16-OH) as white crystalline powder (190 mg, 39%).

M.p. 95–97 °C; R_f = 0.33 ($CHCl_3$/diethyl ether, 1/1, v/v); ^{1}H NMR (400 MHz, $CDCl_3$, 27 °C): δ = 1.24–1.43 (m, 48 H, 2× C≡C$(CH_2)_2$(C$\boldsymbol{H}_2)_{12}$$(CH_2)_2$OH), 1.51–1.61 (m, 8 H, 2× C≡CCH_2C$\boldsymbol{H}_2$$(CH_2)_{12}$ C$\boldsymbol{H}_2$$CH_2$OH), 2.38 (t, $^3J_{H/H}$ = 7.1 Hz, 4 H, 2× C≡CC$\boldsymbol{H}_2$), 3.62 (t, $^3J_{H/H}$ = 6.6 Hz, 4 H, 2× C$\boldsymbol{H}_2$OH), 7.27 ppm (s, 4 H, $C_6\boldsymbol{H}_4$); ^{13}C NMR (100 MHz, $CDCl_3$, 27 °C): δ = 19.60 (C≡C$\boldsymbol{C}H_2$), 25.85 ($\boldsymbol{C}H_2(CH_2)_2$OH), 28.82, 29.03, 29.24, 29.53, 29.61, 29.71, 29.74, 32.93 ($\boldsymbol{C}H_2CH_2$OH), 63.14 ($\boldsymbol{C}H_2$OH), 80.39 ($\boldsymbol{C}$≡CCH_2), 91.93 (C≡$\boldsymbol{C}CH_2$), 123.11 (1,4-C, $\boldsymbol{C}_6H_4$), 131.22 ppm (2,3,5,6-C, $\boldsymbol{C}_6H_4$); MS (70 eV): *m/z* (%): 607 (63) [M^+], 437 (10), 395 (84) [$M^+ - C_{14}H_{28}O$], 381 (85) [$M^+ - C_{15}H_{30}O$], 209 (23), 195 (29), 181 (35), 169 (61), 155 (75), 129 (100); elemental analysis calcd (%) for $C_{42}H_{70}O_2$ (607.00): C 83.10, H 11.63; found: C 82.96, H 11.33.

Hydrogenation reaction: For the hydrogenation of the triple bonds, a procedure described previously [2] was used. Diol (HO-C16pAcPhAcC16-OH, 182 mg, 0.3 mmol) was dissolved in heptane/ethyl acetate/ethanol (100 mL, 3/1/1, v/v/v) and $Pd(OH)_2$ (20% on carbon, 75 mg) was added. The mixture was stirred under hydrogen (10 atm) at room temperature for 18 h. Afterwards, the catalyst was removed by filtration and the solvent was evaporated in vacuo to give the diol 18,18'-(1,4-phenylene)di(octadecan-1-ol) HO-C18pPhC18-OH as white crystalline powder (175 mg, 95%) after recrystallization from heptane.

M.p. 105–106 °C; R_f = 0.28 ($CHCl_3$/diethyl ether, 1/1, v/v); ^{1}H NMR (400 MHz, $CDCl_3$, 27 °C): δ = 1.23–1.34 (m, 56 H, 2× $(CH_2)_2(C\boldsymbol{H}_2)_{14}(CH_2)_2OH$), 1.51–1.59 (m, 8 H, 2× $CH_2C\boldsymbol{H}_2(CH_2)_{14}C\boldsymbol{H}_2CH_2OH$), 2.54 (t, $^3J_{H/H}$ = 7.8 Hz, 4 H, $C\boldsymbol{H}_2C_6H_4C\boldsymbol{H}_2$), 3.62 (t, $^3J_{H/H}$ = 6.7 Hz, 4 H, 2× $C\boldsymbol{H}_2OH$), 7.06 ppm (s, 4 H, $C_6\boldsymbol{H}_4$); ^{13}C NMR (100 MHz, $CDCl_3$, 27 °C): δ = 25.75 ($\boldsymbol{C}H_2(CH_2)_2OH$), 29.44, 29.53, 29.60, 29.62, 29.67, 29.69, 31.60 ($\boldsymbol{C}H_2CH_2C_6H_4CH_2\boldsymbol{C}H_2$), 32.83 ($\boldsymbol{C}H_2CH_2OH$), 35.57 ($\boldsymbol{C}H_2C_6H_4\boldsymbol{C}H_2$), 63.12 ($\boldsymbol{C}H_2OH$), 128.20 (2,3,5,6-C, $\boldsymbol{C}_6H_4$), 140.08 ppm (1,4-C, $\boldsymbol{C}_6H_4$); MS (70 eV): *m/z* (%): 615 (100) [M^+], 597 (16) [$M^+ - H_2O$]; elemental analysis calcd (%) for $C_{42}H_{78}O_2$ (615.06): C 82.01, H 12.78; found: C 81.92, H 12.64.

Phosphorylation and quarternisation: The phosphorylation of the diol HO-C18pPhC18-OH (160 mg, 0.26 mmol) and the subsequent quarternisation were performed according to the synthetic procedures described previously [5].

The final bolalipid 18,18'-(1,4-phenylene)bis{octadec-1-yl[2-trimethylammonio)ethylphosphate]} (PC-C18pPhC18-PC) was obtained in 60% yield (145 mg, 0.15 mmol). R_f = 0.30 ($CHCl_3$/MeOH/NH_3, 10/10/3, v/v/v); ESI-MS: *m/z*: 945.86 $[M + H]^+$, 967.68 $[M + Na]^+$, 1912.84 $[2M + Na]^+$; 979.74 $[M + Cl]^-$; HRMS: Calcd for $C_{52}H_{102}N_2O_8P_2$ $[M + H]^+$ 945.7184, found: 945.7179 (−0.5087 ppm); ^{1}H NMR (400 MHz, $CDCl_3/CD_3OD$, 27 °C): δ = 1.13–1.21 (m, 56 H, 2× $(CH_2)_2(C\boldsymbol{H}_2)_{14}(CH_2)_2O$), 1.43–1.53 (m, 8 H, 2× $CH_2C\boldsymbol{H}_2(CH_2)_{14}C\boldsymbol{H}_2$ CH_2O), 2.44 (t, $^3J_{H/H}$ = 7.8 Hz, 4 H, $C\boldsymbol{H}_2C_6H_4C\boldsymbol{H}_2$), 3.09 (s, 18 H, 6× $C\boldsymbol{H}_3$), 3.46–3.48 (m, 4 H, 2× $NC\boldsymbol{H}_2CH_2O$), 3.72–3.77 (m, 4 H, 2× $OC\boldsymbol{H}_2(CH_2)_{17}$), 4.08–4.14 (m, 4 H, 2× $NCH_2C\boldsymbol{H}_2O$), 6.96 ppm (s, 4 H, $C_6\boldsymbol{H}_4$).

2. Physicochemical investigations

2.1 SANS

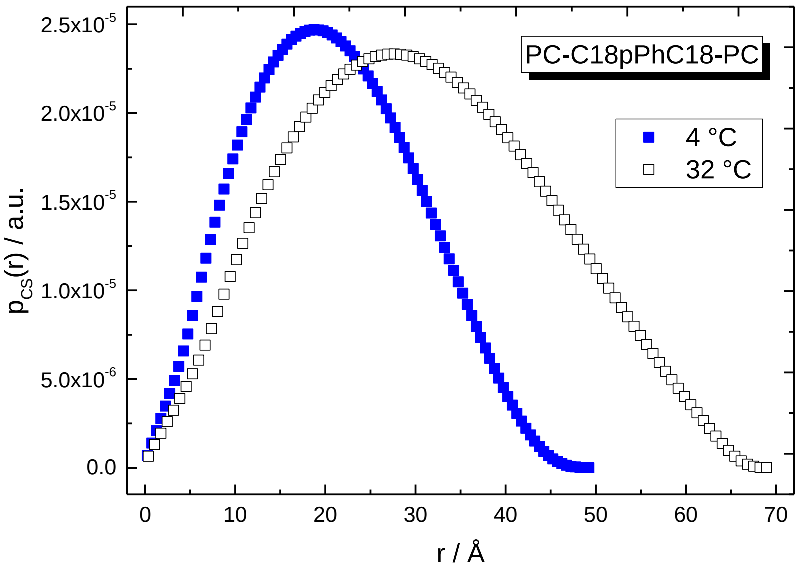

Figure S1: Cross-sectional pair distance distribution function $p_{CS}(r)$ obtained for scattering of a suspension of PC-C18pPhC18-PC (c = 1 mg mL^{-1} in D_2O) at 4 °C (filled blue symbols) and 32 °C (open symbols). IFT analysis has been done in assumption of 2D-objects (infinitely long cylinder).

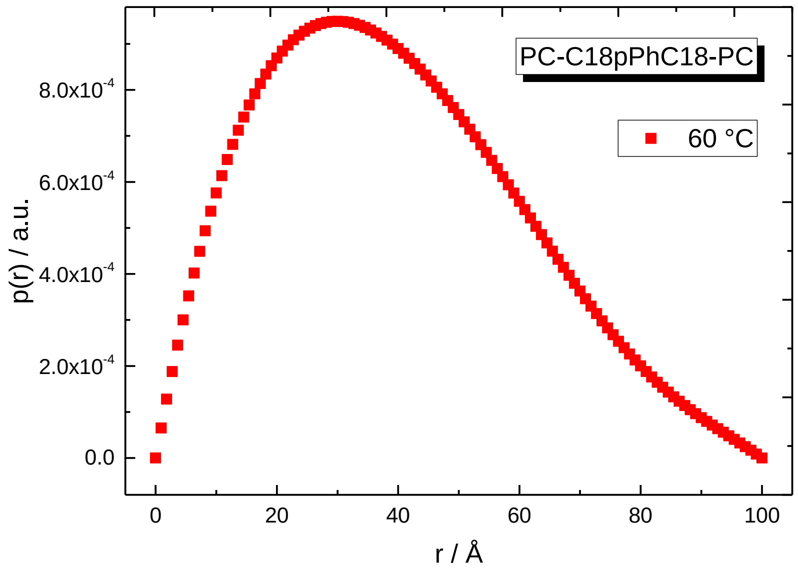

Figure S2: Pair distance distribution function $p(r)$ obtained for scattering of a suspension of PC-C18pPhC18-PC (c = 1 mg mL^{-1} in D_2O) at 60 °C. IFT analysis has been done in assumption of 3D-objects.

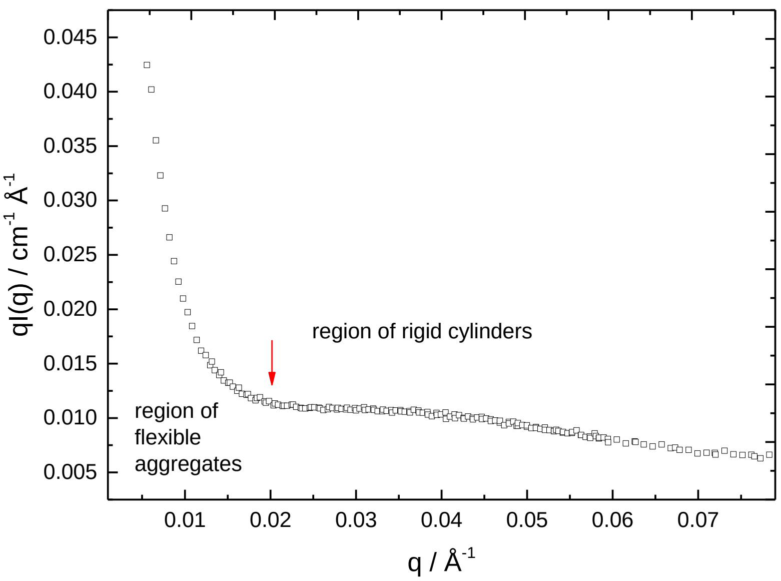

Figure S3: SANS data of a suspension of PC-C18pPhC18-PC (c = 1 mg mL^{-1} in D_2O, at 32 °C) in Holtzer representation.

3. References

1. Eibl, H.; Nicksch, A. 1,3-Propanedeol phosphatides. Ger. Offen. DE 2345057 A1 19750327, **1975**.

2. Drescher, S.; Becker, S.; Dobner, B.; Blume, A. *RSC Adv.* **2012**, *2*, 4052-4054.

3. Drescher, S.; Helmis, K.; Langner, A.; Dobner, B. *Monatsh. Chem.* **2010**, *141*, 339-349.

4. Menger, F. M.; Chen, X. Y.; Brocchini, S.; Hopkins, H. P.; Hamilton, D. *J. Am. Chem. Soc.* **1993**, *115*, 6600-6608.

5. Drescher, S.; Meister, A.; Blume, A.; Karlsson, G.; Almgren, M.; Dobner, B. *Chem. Eur. J.* **2007**, *13*, 5300-5307.
